# Dr. James Magennis

**Published:** 1831-01

**Authors:** 


### OBITUARY.

It is with sincere regret that we record the decease of one of our earliest and ablest contributors,—of Dr. James Magennis, aged seventy-one, who died at his house, Great Chesterford, Essex, on the 30th of October last. Dr. M. was the senior physician in the Navy. His acknowledged writings are contained in the 3d, 4th, 5th, 9th, and 10th volumes of this Journal: these papers are on the Medicinal Effects of Digitalis, on the Cure of Dropsy and Epilepsy, on the Cure of Phthisis Pulmonalis, Observations on Apoplexy, and an article on the Influenza. Some of these communications are very elaborate, particularly those on Digitalis, on Dropsy, and Apoplexy. So highly was Dr. M. esteemed at the place where he resided, that nearly all the inhabitants of the village attended his funeral, to show their last respect to a good man, and a very generous benefactor to the poor.

